# Anterior cervical discectomy and fusion with recombinant human bone morphogenetic protein-2-adsorbed β-tricalcium phosphate granules: a preliminary report

**DOI:** 10.1186/s13018-020-01760-0

**Published:** 2020-07-14

**Authors:** Ze Wang, Soomin Lee, Zheng Li, Shuhao Liu, Qintong Xu, Jian Zhang, Dehua Meng

**Affiliations:** Department of Orthopaedic Surgery, Zhongshan Hospital, Fudan University, Shanghai, 200032 China

**Keywords:** β-tricalcium phosphate, Bone morphogenetic protein-2, Anterior cervical discectomy and fusion

## Abstract

**Background:**

Anterior cervical discectomy and fusion (ACDF) is an alternative to conservative therapy in the treatment of cervical spondylopathy. This study evaluated the clinical outcome of ACDF with BMP-2-adsorbed β-tricalcium phosphate granules.

**Methods:**

Thirty-two patients with cervical spondylopathy received treatment of ACDF with BMP-2-adsorbed β-tricalcium phosphate granules. The clinical outcomes were evaluated with the Japanese Orthopedic Association (JOA) scores and Neck Disability Index (NDI). Meanwhile, the cervical curvature and intervertebral heights were obtained through lateral cervical X-ray films pre- and postoperatively at each interval, and the precision of cervical fusion was assessed by three-dimensional computed tomography scan.

**Results:**

The follow-up averaged 15.2 months (range 13–18). Average JOA scores significantly increased from a preoperative point (7.4 ± 1.2) to each interval after surgery (*P* < 0.05). NDI decreased from preoperative point (43.1 ± 9.0) to each interval after surgery (*P* < 0.05). The angles of cervical curvature and intervertebral heights were improved postoperatively and kept throughout the follow-up period. CT scan demonstrated a fusion rate of 82.9% at 6 months postoperatively and was improved to 100% at 12 months postoperatively. In all cases, no complications appeared and reported due to any lapse in surgical procedure skills throughout the follow-up period**.**

**Conclusions:**

Our preliminary findings suggest that BMP-2-adsorbed β-tricalcium phosphate granules will be an effective alternative to autogenous bone grafting for cervical fusion in treating cervical spondylopathy. Our surgical procedure usingβ-tricalcium phosphate granules could improve neurological function, recover intervertebral height and cervical curvature, and could be potentially exploitable in the clinical setting.

## Background

Anterior cervical discectomy and fusion (ACDF) has been the standard procedure for the treatment of cervical spondylopathy which is non-responsive to conservative therapy. The procedure can decompress the spinal cord and nerve roots and restore the alignment of the spinal column and finally achieve interbody fusion, which may guarantee sustainable clinical outcome [1]. However, an autogenous bone graft is considered to be the “gold standard” for many years [2] and the disadvantages remain including persistent donor site-related pain, potential risk of neurologic impairment and vascular injury, wound infection, and cosmetic problem [3]. Meanwhile, the clinical experience has indicated that there was no upsurge in the successful fusion rate with the corresponding increase in fusion levels [4].

Various researches have proposed many bone replacement or new materials to substitute the autogenous bone graft, but all have its advantages and disadvantages [5]. Allogeneic bone and demineralized bone matrix have been applied to the clinical setting, but due to the lack of resources, possible disease transmission, and the inferior osteogenic capacity they possess may limit the wide usage and replacement of the autogenous bone in interbody fusion. The idea and concept of tissue-engineered scaffolds, cell-based treatment, and gene therapy for spine fusion are still in the groundwork stage and may require more time to be clinically implemented after experimental testing and clinical trials.

The bone morphogenic protein-2 (BMP-2) was detected and purified from demineralized bone and is known to enhance the osteo-inductive properties and promote bone formation without any potential carcinogenic side effects. Synthetic ceramics, β-tricalcium phosphate (β-TCP), is commonly used as a bone substitute in orthopedic surgery and can degrade according to the bone ingrowth. BMP-2 adsorbed β-tricalcium phosphate (TCP) granules prepared by a sustainable combination of both BMP-2 and β-TCP, “the actively artificial bone” as we have mentioned in our study was proved to demonstrate bone conductive and osteo-inductive property and compared favorably with autogenous bone according to interbody fusion performance in vitro or animal experiments [6].

In this clinical study, we evaluated the potential of the actively artificial bone as a substitute for autogenous bone based on a retrospective analysis of the subjects who received the ACDF for the treatment of cervical spondylopathy.

## Methods

### Brief introduction of artificial bone

**Fig. 1:**
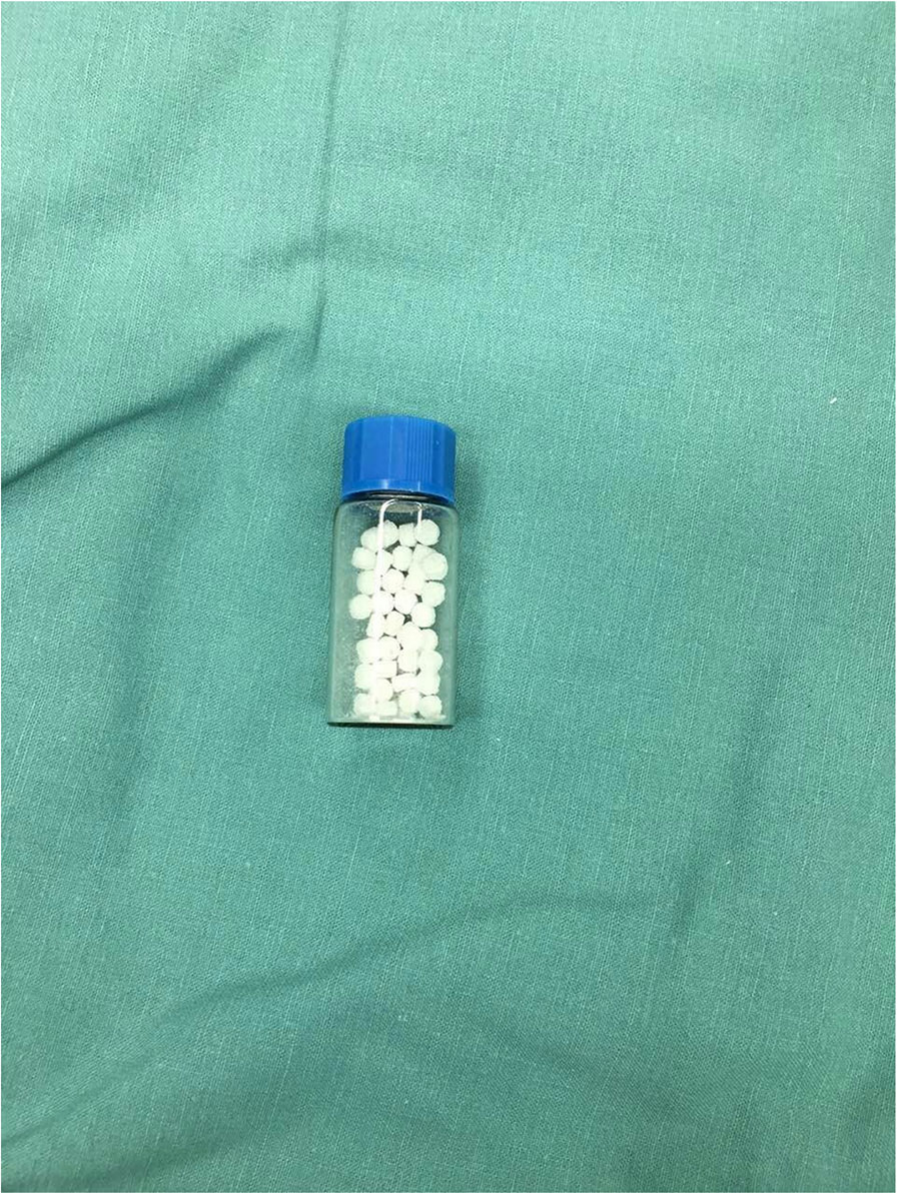
Actively artificial bone

BMP-2-adsorbed β-TCP granules (Rebone, Shanghai Rebone Biomaterial Inc.), we used in our study is a new material processed by the special craft. Its porosity reaches 70%, grain size 300–500 μm. Recombinant BMP2 derived from the *Escherichia coli* system with BMP2 content 1.5 mg/g was used, and the artificial bone was preserved under 4 °C (Fig. 1).

### Patient population

In this retrospective study, 32 consecutive patients with a mean 15.2 months follow-up period (13–18 months) included 19 males and 13 females, aged between 35 and 84 years (mean, 66.2 years). All enrolled patients underwent ACDF with cage (ROI-C, LDR, Troyes, France) packed actively artificial bone during the years 2013 and 2014. Total 82 surgical levels were included: 1 level at 6 cases, 2 at 7 cases, 3 at 14 cases, and 4 at 5 cases from C2 to C7. All patients were treated indicated for symptomatic cervical disc hernia or spondylosis. The patients with ossified posterior longitudinal ligament (OPLL) and those who needed combination with posterior depression were excluded from the study. In addition, revision surgery was beyond the scope of our study. This study was approved by the Institutional Review Broad of Zhongshan Hospital, Fudan University, Shanghai, China. All patients were informed and explained regarding the details and outcome of the grafting procedure and the substitutes we used. The signed informed consents were obtained from all patients before surgical procedures and the patient consent procedure was approved by the ethics committees.

### Surgical procedure

**Fig. 2:**
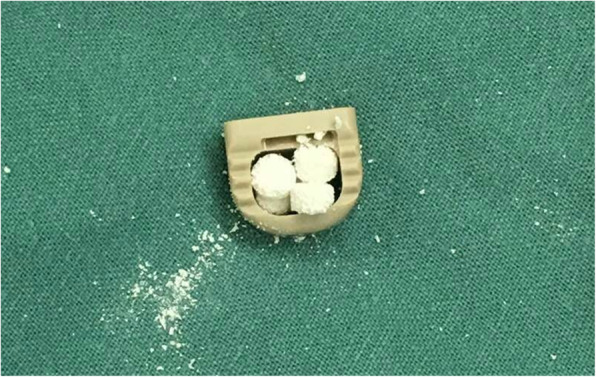
Cage packed with actively artificial bone

All operations were performed by the same surgical team, applying a standard anterior surgical approach. Discectomy was performed, osteophytes compressing the nerve roots, and spinal cord was removed with caution if necessary. The endplates were abraded until the bony part to prevent cage subsidence. The appropriate size for the cage was decided by templating and intraoperative evaluation. The volume of actively artificial bone per level varied based on the cage size, and the cage was packed with an estimated 300–400 mg per level to contact the endplate when inserted into interspace (Fig. 2). After operations, neck collars were used for 6 weeks postoperatively.

### Clinical evaluation

Data from the three parts were used to evaluate the clinical outcome of patients. Neurological function was assessed during the follow-ups at the preoperative time, 3 months, 6 months, 1 year, and the late interval after surgery by the Japanese Orthopedic Association (JOA) scores recording. Recovery of neck disability as well was assessed during the same visits after surgery by Neck Disability Index (NDI) [7]. The patient’s satisfaction towards surgery was also assessed at the final follow-up based on Odom’s criteria [8]. All recording was completed by an independent observer in our clinic. In addition, complications during the perioperative period and after surgery were recorded.

### Radiologic assessments

Patients were evaluated radiographically through the cervical X-ray before surgery, 3 months, 6 months, 12 months after surgery, and at the late follow-up period. To be specific, in cervical X-ray, the cervical curvature angle or the Cobb angles were measured from C2 to C7, drawing a line either parallel to the inferior endplate of C2, and another line parallel to the inferior endplate of C7. Perpendicular lines are then drawn from each of the 2 lines and the angle between the crossing of the perpendicular lines was interpreted as cervical curvature angle [9]. Intervertebral heights of surgical levels were measured by the distance between midpoint at the upper and lower endplate. Thin-cut CT scan with sagittal and coronal reconstructions was obtained at 6 months and 12 months after surgery. All images were collected and evaluated blindfolded by two clinicians and radiologists for interbody fusion by the criteria used by Singh et al*.* [10]. Outcomes of fusion assessment were divided into five parts: definitely fused, probably fused, indeterminate probably not fused, and definitely not fused. The rate of fusion was calculated at last based on the fusion condition of each surgical level.

### Statistical analysis

All data were expressed as mean ± standard deviation, and all comparison was conducted using the paired *t* test. The difference between intervals of follow-ups was considered to be statistically significant when *P* < 0.05.

## Results

### Clinical outcomes

All patients after successful implantation have improved without any severe complications during the perioperative period and after surgery. One patient had a complaint of dysphasia after surgery, relieved without any intervention, and symptoms disappear in the late follow-up. One patient presented with transiently radicular pain and recover at discharge. In addition, there were no device failures and no patients experienced symptom relapse or deterioration. None of the patients needed revision surgery.

The mean JOA score before significantly increased to each interval after surgery, meanwhile the mean NDI score before surgery significantly decreased to each interval after surgery. Besides, two kinds of scores did not oppose during the follow-ups and there is no difference among intervals after surgery. Clinical outcomes based on Odom’s criteria were excellent in 19 patients (26.7%), good in 11 patients (60.0%), fair in one patient, and bad in one patient with an eligibility rate of 93.8% (Table 1).

**Table 1:** The JOA and NDI scores at each follow-ups (*n* = 32, mean ± deviation)

Variable	Before surgery	3 months	12 months	Final follow-up
JOA score	7.4 ± 1.2^a^	10.6 ± 1.7	11.7 ± 1.1	11.0 ± 1.8
NDI score	43.1 ± 9.0^b^	23.8 ± 4.1	19.0 ± 2.7	18.7 ± 1.1

^a^There was statistically significant difference compared to the value before surgery for each follow-up

^b^There was statistically significant difference compared to the value before surgery for each follow-up

### Radiological outcomes

The cervical curvature angles were compared in each interval and a significant improvement was noticed after the surgery. The angles deteriorated slightly at the final follow-up compared to that at 6 months, but there was no statistically significant difference among each follow-up. Intervertebral heights of surgical levels were significantly restored and maintained well, though the heights at 6 months seem to be worse than that at the final follow-up and no statistically significant difference existed (Table 2).

**Table 2:** Cervical curvature angles and intervertebral heights (mean ± deviation)

Index	Before surgery	3 months	6 months	Final follow-up
Angles/*n* = 32, (°)	13.1 ± 3.4^c^	24.4 ± 4.1	23.8 ± 1.6	23.2 ± 2.1
Heights/*n* = 82, (mm)	6.0 ± 1.1^d^	8.5 ± 1.1	8.4 ± 1.4	8.1 ± 1.1

^c^There was statistically significant difference compared to the value before surgery for each follow-up

^d^There was statistically significant difference compared to the value before surgery for each follow-up

**Fig. 3:**
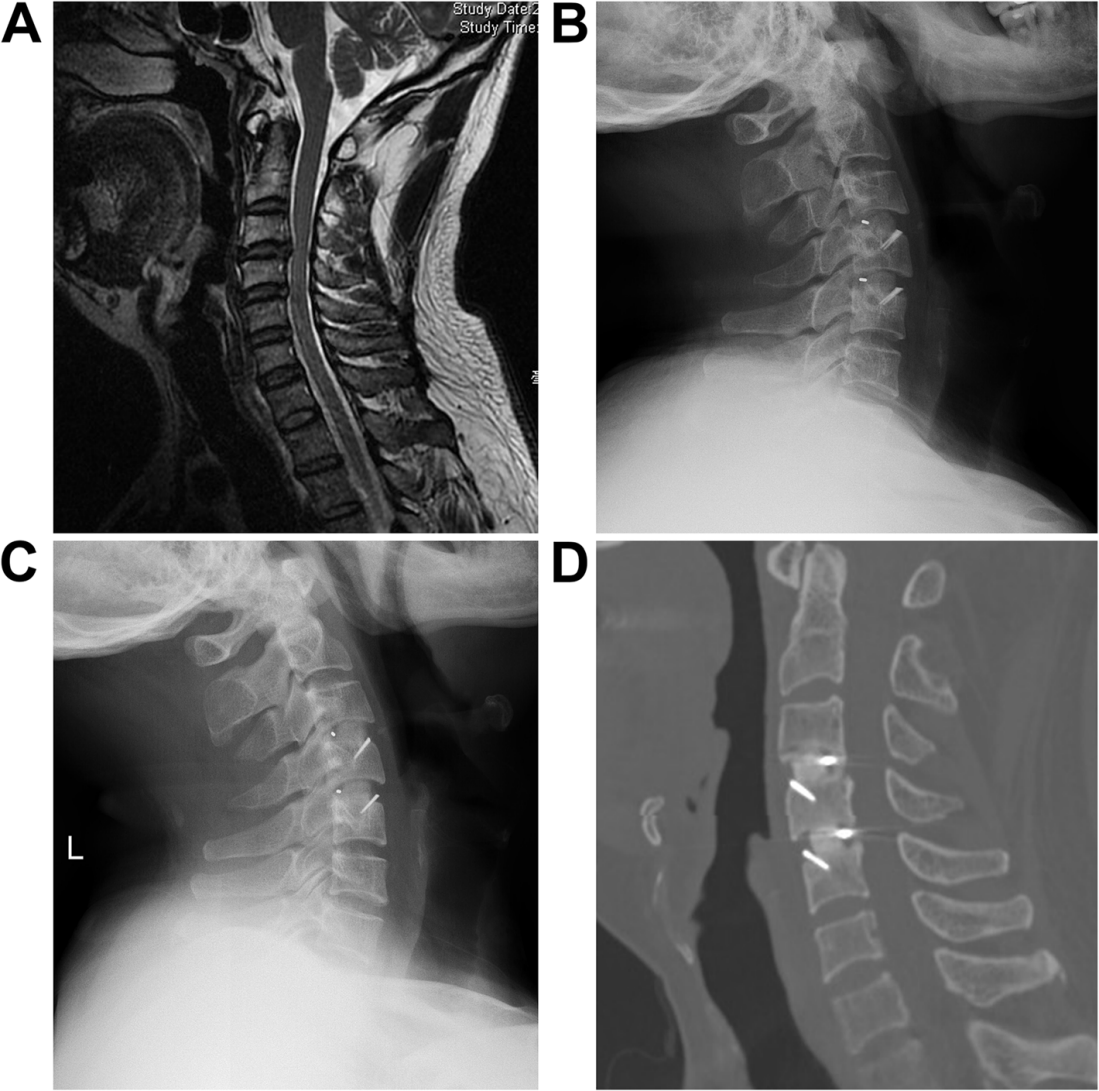
**a** A male patient with cervical spondylosis at contiguous levels. **b** At 3 months after surgery, cage was located well, and cervical curvature was kept good. **c** At 6 months, curvature angle and intervertebral heights remained well. **d** At 6 months, the sagittal reconstruction demonstrated solid interbody fusion

As for the rate of interbody fusion, 62.4% (45/82) of the levels at 6 months after surgery were categorized as definitely fused with a total rate of 97% as definitely and probably fused (68/82). And at 12 months after surgery, 93.1% (77/82) of the levels were categorized as definitely fused with a total rate of 100% as definitely and probably fused (82/82) (Fig. 3).

## Discussion

Various kinds of calcium phosphate ceramics with quality of low crystallinity and porous structure have been tested as substitutes in several basic types of research and clinical settings of orthopedic surgery, which had similar elements and structures compared to natural bone [11]. In light of suitable porosity and grain size, β-TCP showed superior properties in bone conductivity and osteo-conduction and displayed good biocompatibility [12]. Yuan et al*.* [13] reviewed HA, TCP, and related ceramics and concluded thatβ-TCP is the most osteo-inductive and also with excellent degradation in vivo. After proper implantation at the target site, β-TCP functions as a material characteristic of absorption and interpose to separate soft tissue, effectively induce recipient bone formation, and was replaced perfectly at last with an appropriate time period [13]. However, the ideal structure for biological activity was questioned as the mechanical stiffness between cortical and cancellous bone can hardly bear the pressure alone without proper and complete internal fixation in spine fusion. Meanwhile, the lack of osteo-inductive agents matched well with the autogenous bone when fusion outcome was judged.

Bone morphogenetic proteins (BMPs) belong to a subfamily of the transforming growth factor-β and play an important role in inducing cartilage and bone formation [14]. BMP-2 has been studied the most, but the agent is easily degraded and absorbed quickly which makes it hard to control the process as anticipated [15]. Though the US Food and Drug Administration has approved the commercial use of BMP2-absorbed collagen or gelatin for lumbar fusion procedures, problems remain with the use of ideal carriers. Packing interspace or cage with BMP-2, dissolved in a complicated procedure, may prolong the operation time and is hard to standardize the dosage used [16, 17]. In addition, the specification of carriers also will decide the rate and spectrum of BMP2 release that can influence the fusion effect and emergence of any complications.

In our study, the BMP-2-adsorbed β-TCP is processed by a special craft, in order to make up the defects when each part is applied alone. β-TCP, as a delivery system, can control the release of BMP and could retain as a barrier to interpose between bone tissue and graft. On the other hand, BMP2 resulted in better osteo-conduction that could induce mesenchymal migrate into the material and activate cells to proliferate and differentiate into osteoblasts, meanwhile degrading the material to balance the material absorption and bone ingrowth. Cao et al. [18] have reported that BMP-2-adsorbed β-TCP granules repaired the femoral defects of rabbits. They have also observed that a lot of woven bone and bone trabecula with marrow was detected at 8 weeks and the release of rhBMP-2 from the material was slower and smoother than the control group, which was considered as the real physiological state. Dohzono et al. [19] also found that BMP-2-adsorbed β-TCP granules could achieve similar posterolateral lumbar fusion compared to autogenous bone graft based on radiographical, histological, and biomechanical evaluation in a rabbit model. Moreover, they have also suggested that the bone mass-produced was highly dependent on the dosage. In our knowledge, it is the first time that BMP-2-adsorbed β-TCP granules were applied to anterior cervical surgery for interbody fusion in patients with cervical spondylopathy. We found that the cervical curvature and intervertebral height were significantly improved compared to preoperative condition and sustained through follow-up, which means artificial bone could play a significant role in interbody fusion and eventually preserve the physiological alignment of the cervical spine. The CT scan demonstrated similar results showing that most of the operative levels have reached solid fusion at 6 months postoperatively and all patients had the complete fusion process. The fusion rates and efficacy of complete fusion in the artificial bone group were not inferior to that of the autogenous bone group, though the fusion rate mentioned in other studies was lower than that in the autogenous bone group at 6 months. Maybe, this discrepancy might have been due to the criteria used in our evaluation of CT images [20]. Besides, we also found that cervical alignment and intervertebral height deteriorated as the time advanced, but the difference was not statistically significant. In our opinion, various factors have influenced, such as the age, type of work, the cervical vertebra number and the position of level we fused, the lifestyle before and after the operation, and adjacent segment disease apart from the nature of the material and the surgical technique we have followed. Meanwhile to our satisfaction, everyone gained a desirable effect based on JOA and NDI scores. Nevertheless, as we know, the early solid fusion is the prime goal by which ACDF could guarantee the long-term relief of symptoms. So, we would continue to extend the follow-up of the patients in order to inspect the long-term clinical outcome and potential loss of postoperative intervertebral height and alignment. For complications such as neck swelling, ectopic bone formation, and dysphagia, we agree to the Owens point with caution that technical modifications can minimize the occurrence of complications. Accordingly, on the assessment of complications, the fusion enhanced by BMP2 was consistent with autogenous bone fusion [21].

Our reported retrospective study has its own inherent drawbacks, and with the limited sample size and the inadequate following time, the study could hardly derive any convincing conclusion. Moreover, the radiographical judgment is biased by the internal fixation and cage applied in our series.

## Conclusions

As a whole, this preliminary report suggests that BMP-2-adsorbed β-tricalcium phosphate granules could be a potential alternative to autogenous bone grafting for cervical fusion in the treatment of cervical spondylopathy, and may help to improve neurological functions and recover intervertebral height and cervical curvature in patients.

## Data Availability

The datasets used and/or analyzed during the current study are available from the corresponding author on reasonable request.

## Abbreviations

β-TCP: β-tricalcium phosphate
ACDF: Anterior cervical discectomy and fusion
BMP-2: Bone morphogenic protein-2
BMPs: Bone morphogenetic proteins
JOA: Japanese Orthopedic Association
NDI: Neck Disability Index
OPLL: Ossified posterior longitudinal ligament
TCP: Tricalcium phosphate
